# Cognitive impairment and health-related quality of life amongst older Australians: evidence from a longitudinal investigation

**DOI:** 10.1007/s11136-023-03449-3

**Published:** 2023-06-08

**Authors:** Syed Afroz Keramat, Vanessa Lee, Rajat Patel, Rubayyat Hashmi, Tracy Comans

**Affiliations:** 1 Faculty of Medicine, Centre for Health Services Research, The University of Queensland, Brisbane, QLD Australia; 2 Faculty of Medicine, The University of Queensland, Brisbane, QLD Australia; 3 The Australian Centre for Housing Research, The University of Adelaide, Adelaide, Australia

**Keywords:** Cognitive impairment, BDS, SDMT, HRQoL, PCS, MCS, SF-6D

## Abstract

**Introduction:**

Australia’s population is steadily growing older, with older persons expected to make up over 20% of the population by 2066. Ageing is strongly associated with a significant drop in cognitive ability, ranging from mild cognitive impairment to severe cognitive impairment (dementia). This study examined the association between cognitive impairment and health-related quality of life (HRQoL) in older Australians.

**Methods:**

Two waves of longitudinal data from the nationally representative Household, Income and Labour Dynamics in Australia (HILDA) survey were utilised, with the age cut-off for older Australians defined as above 50. The final analysis included 10,737 person-year observations from 6892 unique individuals between 2012 and 2016. This study utilised the Backwards Digit Span (BDS) test and Symbol Digit Modalities test (SDMT) to assess cognitive function. HRQoL was measured using the physical and mental component summary scores of the SF-36 Health Survey (PCS and MCS). Additionally, HRQoL was measured using health state utility values (SF-6D score). A longitudinal random-effects GLS regression model was used to analyse the association between cognitive impairment and HRQoL.

**Results:**

This study found that approximately 89% of Australian adults aged 50 or older had no cognitive impairment, 10.16% had moderate cognitive impairment, and 0.72% had severe cognitive impairment. This study also found that moderate and severe cognitive impairment were both negatively associated with HRQoL. Older Australians with moderate cognitive impairment scored worse on the PCS (β = − 1.765, SE = 0.317), MCS (β = − 1.612, SE = 0.326), and SF-6D (β = − 0.024, SE = 0.004) than peers without cognitive impairment given other covariates reference categories remain constant. Older adults experiencing severe cognitive had lower PCS (β = − 3.560, SE = 1.103), and SF-6D (β = − 0.034, SE = 0.012) scores compared to their counterparts with no cognitive impairment given other covariates reference categories remain constant.

**Conclusion:**

We found evidence that HRQoL is negatively associated with cognitive impairment. Our findings will be beneficial for the future cost-effectiveness intervention targeted at reducing cognitive impairment since it provides information on the disutility associated with moderate and severe cognitive impairment.

## Introduction

With increasing age, the susceptibility to age-associated cognitive impairment increases [1]. Concerningly, the demographic profile of the world population is shifting towards older age with improved life expectancy. As of 2020, older Australians (aged ≥ 65 years) comprised 16% of the total Australian population and is projected to increase to 20.7% by 2066 [2]. This unprecedented growth of population ageing presents significant challenges relating to healthcare, independence, social, and community interaction among the elderly.

Ageing is associated with cognitive impairment ranging from mild impairment to dementia (severe impairment). Such impairment is a major cause of dependency and disability in the elderly. The age at which cognitive abilities decline is subject to debate [3–6]. However, longitudinal data has shown cognitive decline is evident at all ages between 45 and 70 years, with an accelerated decline in the oldest age groups [7]. Amongst older Australians (aged ≥ 65 years), the estimated prevalence of cognitive impairment ranges from 7.7 to 33.3% [8, 9]. Globally, the prevalence of cognitive impairment in those aged ≥ 50 years, ranges from 5.1 to 41.0%, with a median prevalence of 19.0% [10]. It is estimated that the prevalence of severe cognitive impairment will be 82 million in 2030, and will rise to 152 million by 2050 [11, 12].

There is well-established literature on the vital role that cognitive ability occupies in an individual’s daily functioning. Cognitive ability is defined as “the skills involved in performing the tasks associated with perception, learning, memory, understanding, awareness, reasoning, judgment, intuition, and language” [13]. The degree of cognitive impairment may range from mild deficits in memory, language, executive functioning, or visuospatial capabilities [14] to clinically significant deficits associated with other pathologies, such as dementia and Alzheimer’s disease [15, 16]. Given the complex multitude of influences on cognitive impairment, a multi-dimensional measure of health-related outcomes is needed to assess health and well-being.

HRQoL is a multi-dimensional construct that encompasses physical, mental, emotional, and social functioning [17]. It provides a broad summary of overall health status by incorporating elements that have been shown to affect health. Self-reported health outcomes can be measured accurately using generic non-preference and preference-based quality of life instruments, allowing for meaningful comparisons between healthy and clinical populations [18]. However, it is argued that condition-specific preference-based measurements may be more successful than a generic preference-based measure of HRQoL because they are more responsive to a particular health condition [19].

The association between cognitive impairment and HRQoL has been previously investigated, yielding conflicting findings. Notably, a number of these studies were undertaken in specific age groups and settings. Cognitive impairment has been associated with lower HRQoL in the Chinese (utilised EQ-5D) [20], Swedish (utilised EQ-5D) [21], and Turkish (utilised CDC HRQOL-4) [22] elderly. Other studies have shown similar findings in older adults with ailments, such as Alzheimer’s disease (utilised Quality of Life–Alzheimer’s Disease Scale) [23], dementia (utilised Qualidem) [24], and neurological disease [25]. Conversely, other evidence suggests that HRQoL is not impacted by cognitive impairment in nursing home residents (utilised Nursing Home Vision-Targeted Health-Related Quality of Life Questionnaire, VF-14, and the SF-36) [26], dementia patients [27] or institutionalized older Canadians (utilised EQ-5D-3L) [28]. One study of older Belgian adults found that lower HRQoL (using Alzheimer's Disease Related Quality of Life) was associated with dementia, rather than mild cognitive impairment (MCI) [29]. These inconsistent findings may be attributed to the subjective nature of HRQoL, instruments used, statistical methods and settings. Thus, a reliable comparison of findings across studies is challenging. Further, research on the complex interaction between degrees of cognitive impairment and HRQoL and how it evolves over time is needed across a broader spectrum of older age. This calls for further longitudinal research to elucidate how cognitive impairment impacts HRQoL over time.

The unprecedented proportional population growth of the elderly, in conjunction with the negative association between cognitive impairment and HRQoL, will pose significant logistical and financial healthcare challenges in the future. It is thus paramount that further research be conducted into developing medical treatments and funding preventative public health measures to address the cognitive decline. The current study will examine the association between cognitive impairment and HRQoL among older Australians (≥ 50 years old) using longitudinal data from the Household, Income and Labour Dynamics (HILDA) survey. Understanding the association between cognitive impairment and HRQoL may assist in identifying effective measures to support healthy ageing. It may also serve to optimize how resources are distributed into conserving and/or relieving cognitive impairment symptoms and subsequently, improving and maintaining HRQoL amongst the elderly.

## Methods

### Data and sample selection

This study sourced data from the Household, Income and Labour Dynamics in Australia (HILDA) survey. The survey collects annual data from a nationally representative cohort of Australian adults and has been running since 2001. It follows over 17,000 Australian adults over the course of their lifetimes and collects information on a variety of aspects of daily life, including but not limited to: household and family dynamics, labour and income statistics, education levels, and health outcomes. A more thorough guide for understanding HILDA data and documentation can be found elsewhere [30].

**Fig. 1:**
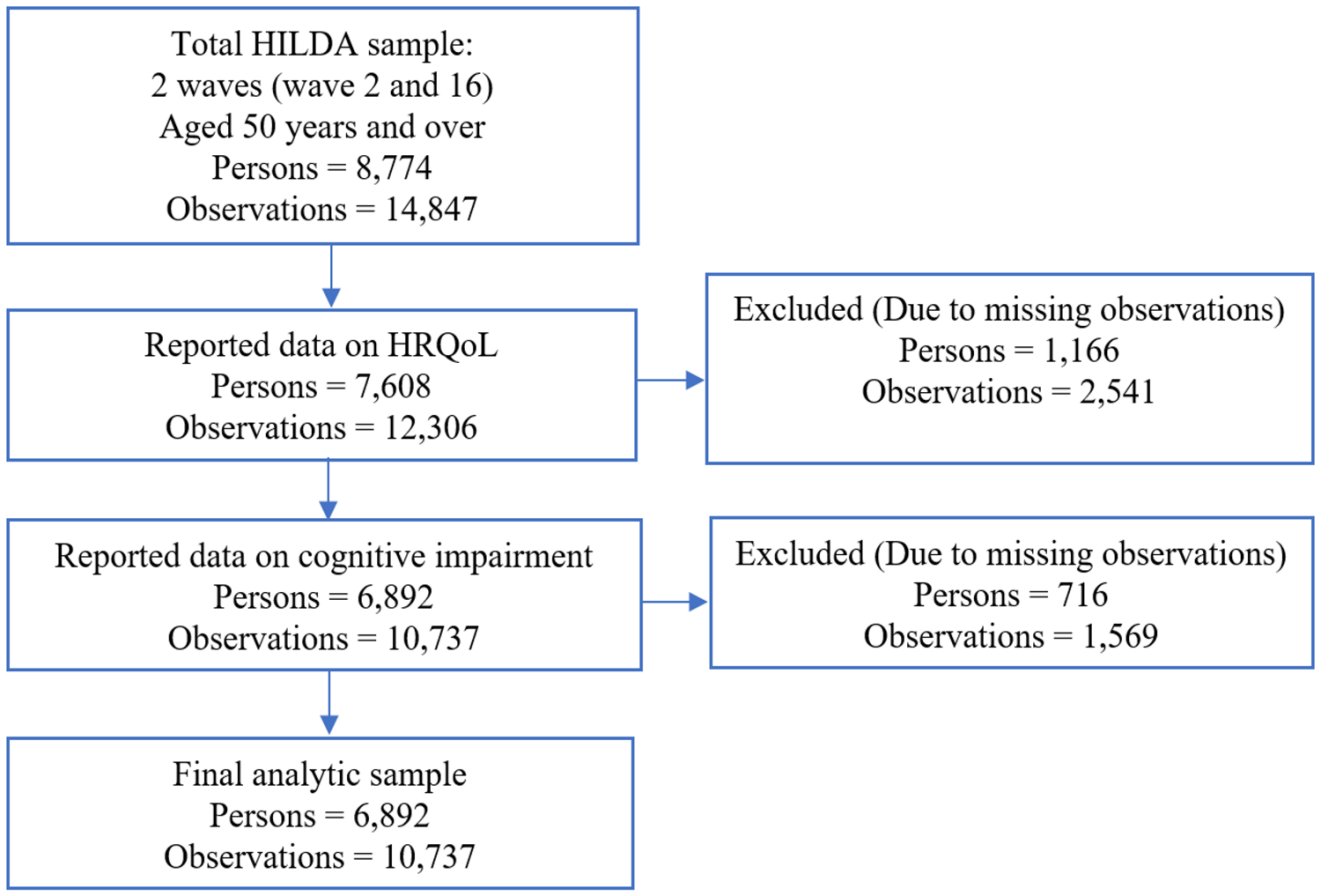
Participants flow into the analytic sample and missing data

The data sourced for this study were extracted from wave 12 (2012) and wave 16 (2016), as these were the only waves where the survey collected information on cognitive impairment. Wave 12 was considered the baseline and wave 16 was considered the follow-up. This study was limited to older Australians, so only observations from those aged 50 years or above were included. Participants with missing data on the exposure variables (cognitive function test outcomes) and the outcome variables (HRQoL outcomes) were excluded. This resulted in a dataset of 10,737 person-year observations from 6,892 unique individuals. The sample selection, including exclusion criteria and breakdown of missing observations, is outlined in Fig. 1.

### Dependent variables

The HILDA survey records HRQoL data through RAND Corporation’s 36-Item Short Form Survey (SF-36). This questionnaire consists of 36 generic and easily administered questions that span 4 physical dimensions and 4 mental dimensions of health [31]. Participant responses are scored from 0 to 100 to represent the worst and best health status for each specific dimension of health. The values of the 8 dimensions of health are then crystallized into two summary component scores: the physical component summary (PCS) score and the mental component summary (MCS) score. The PCS and MCS scores were calculated using the recommended scoring algorithm for Australians [32] and standardized using a linear z-score transformation with a mean of 50 and a standard deviation (SD) of 10. The final PCS and MCS scores in this analysis ranged from 6.04 to 72.14 and 4.36 to 72.79, respectively.

Notably, while the SF-36 is a fairly comprehensive measure of health status, it does not consider utility, which is the measure of preference that an individual imparts onto a particular health state. It equally values each health dimension regardless of how any individual HILDA participant may prioritize aspects of their health. To rectify this, HILDA also incorporates the SF-6D, a derivative of the SF-36 that generate utilities and thus is more econometrically valuable [33]. The SF-6D ranges from 0.29 to 1, where 1 represents full health and 0.29 indicates worst health state.

### Predicting variable

Cognitive ability amongst participants is reported by HILDA through the use of previously established cognitive function tests; they are easily administered and thus are compatible with the face-to-face survey design employed by HILDA. This study uses the Backwards Digit Span test (BDS) and the Symbol Digit Modalities Test (SDMT) as the relevant markers for cognitive function. These particular tests have also seen previous use in identifying cognitive impairment in multiple sclerosis patients [34, 35] and acutely hospitalized patients [36]. The BDS is a test wherein the participant is given a string of digits and is asked to repeat them backwards [37]. The BDS assesses working memory and is scored from 0 to 8. The SDMT is a test where the participant pairs specific numbers with random geometric figures [38]. The SDMT assesses central processing and is scored from 0 to 110.

For this study, previously developed criteria were used to determine the threshold for cognitive impairment: participants who score ≥ 1.0 standard deviations (SD) below the mean on either the BDS or SDMT or both are considered to have MCI, and participants who score ≥ 1.5 SD below the mean for both tests are considered to have severe cognitive impairment [39]. This resulted in the cut-off for the BDS being ≤ 3, and the cut-off for SDMT being ≤ 30, such that any participant scoring at or below the cut-off for either test is considered mildly cognitively impaired. Any participant scoring ≤ 2 on the BDS and ≤ 24 on the SDMT tests will be considered to have severe cognitive impairment.

### Control variables

A set of socio-demographic and health-related characteristics, along with health-related behaviours, were considered as potential confounders for this study. The variables include age, gender, relationship status, highest education level completed, household annual disposable income, labour force status, Indigenous status, region of residence, Body Mass Index (BMI), disability, smoking status, alcohol consumption, and physical activity. A categorical breakdown of these variables is found in Table 1.

**Table 1:** Control variables list and description

Variable	Measure
Socio-demographic characteristics	
Age	0 = 50–64 years 1 = ≥ 65 years
Gender	0 = Male 1 = Female
Relationship status	0 = Single (never married and not living with someone, separated, divorced, and widowed) 1 = Couple (married, and living with someone)
Highest education level completed	0 = Year 12 and below (year 12, and ≤ year 11) 1 = certificate courses (diploma, and certificate III/IV) 2 = university degrees (masters or doctorate, graduate diploma, and honours)
Household annual disposable income	0 = Quintile 1 (poorest) 1 = Quintile 2 (poorer) 2 = Quintile 3 (middle) 3 = Quintile 4 (richer) 4 = Quintile 5 (richest)
Labour force status	0 = Employed 1 = Unemployed or not in the labour force (NLF)
Indigenous status	0 = Not of Indigenous origin 1 = Indigenous origin (Aboriginal, Torres Strait Islander, and/or both)
Health-related characteristics	
Weight category	0 = Underweight (BMI < 18.50) 1 = Healthy weight (BMI 18.50–24.99) 2 = Overweight (BMI 25.00–29.99) 3 = Obese (BMI ≥ 30)
Long-term health condition or disability	0 = No 1 = Yes
Health-related behavioural characteristics	
Smoking status	0 = Non-smoker (never smoked, and former smoker) 1 = Current smoker (smokes daily, smokes at least weekly, and smokes less often than weekly)
Alcohol consumption	0 = Non-drinker (never drunk, and ex-drinker) 1 = Current drinker (only rarely, 1–2 days, 2–3 days, 3–4 days, 5–6 days per week and every day)
Physical activity	0 = Less than the recommended level (not at all, less than once, 1 to 2, and 3 times a week) 1 = Recommended level (> 3 times a week and every day)

Note: 1. The study used a ‘modified OECD’ equivalence scale to measure equivalised household annual disposable income

### Analytic strategy

We constructed an unbalanced longitudinal data set consisting of 10,737 person-year observations from 6,892 unique individuals. The subsequent statistical analysis reported the descriptive statistics using mean and standard deviation (SD) for continuous variables, and percentages for categorical variables. The dependent variables of this study are continuous and we fit a longitudinal random-effects regression model to explain the relationship between cognitive impairment and HRQoL. The use of random-effects regression will help to identify the between-person differences in the association between cognitive impairment and HRQoL. The functional form of the panel random-effects regression is as follows.

1 $${\text{HRQoL}}_{it} \, = \,\alpha \, + \,\beta_{1} C_{it} \, + \,\beta X^{\prime}_{it} \, + \,\mu_{i} \, + \,\varepsilon_{it}$$

$\mathrm{HRQoL}$ denotes the outcome variables: two summary measures (PCS and MCS), and health utility index (SF-6D) derived from the SF-36 questionnaire. C indicates the main variable of interests (cognitive impairment). $X^{\prime}_{it}$ refers to the vector of time invariant and time-varying control variables. $\alpha$ is the model’s grand intercept, $\beta$ is the model parameter of interests to be estimated, and *β*′ indicates vector of co-efficient. $\upmu$ represents individual-specific component that are constant over time and $\upvarepsilon$is the time and person-specific error term that is presumed to have no correlation with the regressors. The subscripts i and t in Eq. 1 represent individual and time, respectively.

All models are adjusted by age, gender, relationship status, highest education level completed, household annual disposable income, labour force status, Indigenous status, region of residence, BMI, disability status, smoking status, alcohol consumption, and physical activity. A p-value < 0.05 was considered significant, with p-values of < 0.01 and < 0.001 also reported for greater evidence of significance. The statistical analysis was performed using Stata version 17.0 (Stata SE 17, College Station, TX: StataCorp LLC, USA).

## Results

### Descriptive analyses

Table 2 summarizes the study population’s socio-demographic, health-related characteristics, and health-related behaviours in the baseline wave (2012), final wave (2016), and pooled waves (2012 to 2016). The pooled data showed that 41% of participants were over 65 years old, slightly more than half were female (53%), almost two-thirds were in a relationship (65%), almost a quarter had completed a university degree (24%), slightly more than half were unemployed or retired (52%), nearly 2% identified Indigenous, 36% resided in a regional or remote area, 29% were obese, 42% have some form of disability, 13% were current smokers, 81% were current drinkers, and one-third of the respondent performed the recommended level of physical activity (33%).

**Table 2:** Distribution of the analytic sample (socio-demographic and health-related characteristics): Baseline, final wave, and pooled data

Variables	Baseline wave (2012)		Final wave (2016)		Pooled sample (2012 and 2016)	
	n	%	n	%	n	%
Socio-demographic characteristics						
Age						
50–64 years	3,049	58.72	3,247	58.56	6,296	58.64
≥ 65 years	2,143	41.28	2,298	41.44	4,441	41.36
Gender						
Male	2,436	46.92	2,624	47.32	5,060	47.13
Female	2,756	53.08	2,921	52.68	5,677	52.87
Relationship status						
Single	1,813	34.92	1,970	35.53	3,783	35.23
Couple	3,379	65.08	3,575	64.47	6,954	64.77
Highest education level completed						
Year 12 and below	2,279	43.89	2,222	40.07	4,501	41.92
Certificate courses	1,710	32.94	1,947	35.11	3,657	34.06
University degrees	1,203	23.17	1,376	24.82	2,579	24.02
Household annual disposable income						
Quintile 1 (Poorest)	1,039	20.01	1,109	20	2,149	20.01
Quintile 2 (Poor)	1,038	19.99	1,109	20	2,146	19.99
Quintile 3 (Middle)	1,039	20.01	1,109	20	2,148	20.01
Quintile 4 (Richer)	1,038	19.99	1,109	20	2,148	20.01
Quintile 5 (Richest)	1,038	19.99	1,109	20	2,146	19.99
Labour force status						
Employed	2,451	47.21	2,654	47.86	5,105	47.55
Unemployed or NLF	2,741	52.79	2,891	52.14	5,632	52.45
Indigenous status						
Not of Indigenous origin	5,097	98.17	5,434	98.0	10,531	98.08
Indigenous origin	95	1.83	111	2.0	206	1.92
Region of residence						
Major city	3,337	64.27	3,501	63.14	6,838	63.69
Regional or remote area	1,855	35.73	2,044	36.86	3,899	36.31
Health-related characteristics						
BMI						
Underweight	73	1.41	65	1.17	138	1.29
Healthy weight	1,699	32.72	1,748	31.52	3,447	32.10
Overweight	1,994	38.41	2,060	37.15	4,054	37.76
Obese	1,426	27.47	1,672	30.15	3,098	28.85
Disability						
No	2,967	57.15	3,279	59.13	6,246	58.17
Yes	2,225	42.85	2,266	40.87	4,491	41.83
Health-related behaviours						
Smoking status						
Non–smoker	4,544	87.52	4,846	87.39	9,390	87.45
Current smoker	648	12.48	699	12.61	1,347	12.55
Alcohol consumption						
Non–drinker	956	18.41	1,040	18.76	1,996	18.59
Current drinker	4,236	81.59	4,505	81.24	8,741	81.41
Physical Activity						
Less than the recommended level	3,477	66.97	3,760	67.81	7,237	67.40
Recommended level	1,715	33.03	1,785	32.19	3,500	32.60

Note: 1. In the pooled waves, a total of 10,737 person-year observations were considered from 6892 unique persons

Table 3 summarizes the distribution of the exposure variable (BDS and SDMT scores, cognitive impairment level) and outcome variables (SF-36 domain scores, PCS score, MCS score, SF-6D score) across the baseline, final, and pooled across all waves. The outcome measures for the pooled sample are as follows: the mean PCS score was 44.63 (SD = 11.30), the mean MCS score was 50.51 (SD = 10.10), the mean SF-6D score was 0.74 (SD = 0.13). The distribution for the exposure measures in the pooled samples are as follows: the mean BDS score was 4.83 (SD = 1.40), the mean SDMT score was 42.63 (SD = 11.88), 89% were not cognitively impaired, 10.16% were moderately cognitively impaired, 0.72% were severely cognitively impaired.

**Table 3:** Distribution of subjective health scores, test scores, and cognitive impairment: baseline, final wave and pooled data

Variables	Baseline wave (2012)		Final wave (2016)		Pooled sample (2012 and 2016)	
	n	Mean (SD)	n	Mean (SD)	n	Mean (SD)
SF-36 domain scores						
Physical functioning	5,192	74.22 (25.12)	5,545	75.29 (24.77)	10,737	74.77 (24.94)
Role physical	5,192	68.51 (41.37)	5,545	68.73 (41.27)	10,737	68.63 (41.32)
Role emotional	5,192	81.43 (34.84)	5,545	81.54 (34.66)	10,737	81.48 (34.75)
Social functioning	5,192	80.99 (24.44)	5,545	80.29 (24.86)	10,737	80.63 (24.66)
Mental health	5,192	76.62 (16.79)	5,545	76.01 (17.21)	10,737	76.30 (17.01)
Vitality	5,192	60.53 (20.22)	5,545	59.90 (20.48)	10,737	60.20 (20.36)
Bodily pain	5,192	65.84 (24.79)	5,545	65.42 (24.82)	10,737	65.62 (24.80)
General health	5,192	63.97 (21.66)	5,545	63.68 (21.67)	10,737	63.82 (21.67)
SF-36 component summary scores						
PCS	5,192	44.49 (11.34)	5,545	44.75 (11.26)	10,737	44.63 (11.30)
MCS	5,192	50.74 (10.01)	5,545	50.30 (10.17)	10,737	50.51 (10.10)
Utility score						
SF-6D	5,192	0.74 (0.13)	5,545	0.74 (0.12)	10,737	0.74 (0.13)
Backward Digit Span (BDS) test score	5,192	4.80 (1.40)	5,545	4.87 (1.40)	10,737	4.83 (1.40)
Symbol Digit Modalities Test (SDMT) score	5,192	42.02 (12.01)	5,545	43.20 (11.73)	10,737	42.63 (11.88)
Cognitive impairment, n (%)						
No	4,559	87.81	5,010	90.35	9,569	89.12
Moderate	597	11.50	494	8.91	1,091	10.16
Severe	36	0.69	41	0.74	77	0.72

Note: 1. In the pooled waves, a total of 10,737 person-year observations were considered from 6892 unique persons

**Fig. 2:**
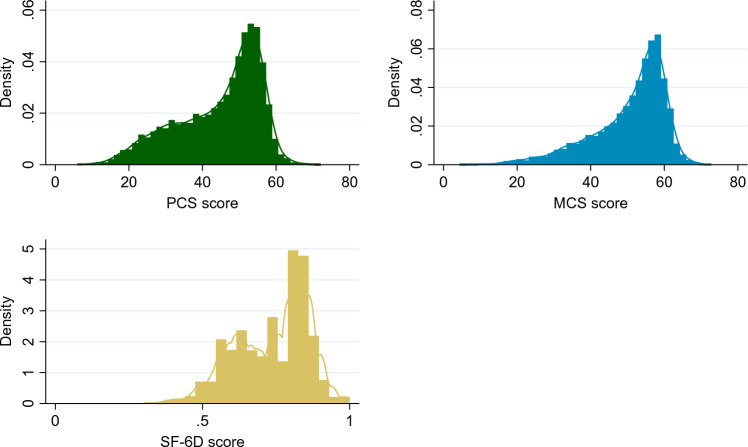
Distribution of PCS and MCS scores and SF-6D utility values

The distribution of the primary outcome measures for the study population is shown in Fig. 2. The PCS scores ranged from 6.0 to 72.1, and the MCS scores ranged from 4.4 to 72.8. Majority of participants had PCS and MCS scores between 40 and 60, with a smaller percentage scoring below 40 and above 60, indicating a right-skewed distribution. The SF-6D utility scores are right skewed similarly to the PCS and MCS scores. The SF-6D scores range from 0.30 to 1, with majority of participants scoring between 0.75 and 1.

**Fig. 3:**
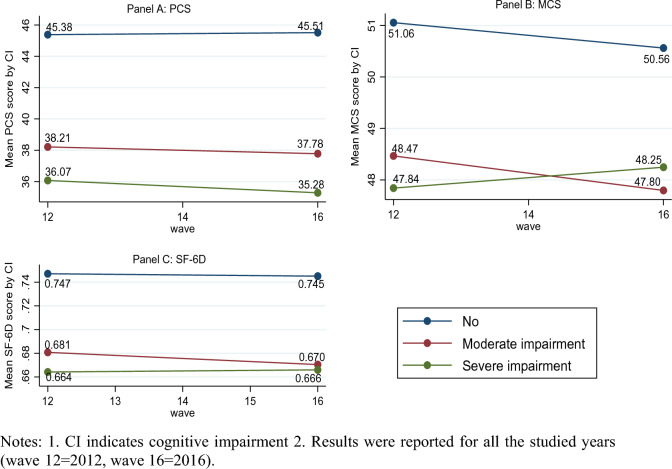
Mean PCS, MCS, and SF-6D score by cognitive impairment

Figure 3 shows the trend of PCS, MCS, and SF-6D scores of the study population over the 4-year observation period between 2012 and 2016, stratified by level of cognitive impairment. The figure shows that the PCS (Panel A), and SF-6D (Panel C) scores for those who were moderately or severely cognitively impaired were much lower than those with no cognitive impairment in all waves. For instance, the mean PCS scores of adults with no cognitive impairment, moderate cognitive impairment and severe cognitive impairment are 45.51, 37.78, and 35.28, respectively in the final wave (wave 16). Figure 3 also demonstrates that the decline in SF-6D is more pronounced in those who are moderately (0.670) and severely (0.666) cognitively impaired compared to those who were not cognitively impaired (0.745) in wave 16. We observed that the MCS scores for those who were moderately and severely cognitively impaired were lower than those with no cognitive impairment (Panel B). The mean MCS scores of adults with no cognitive impairment, moderate cognitive impairment and severe cognitive impairment are 50.56, 47.80, and 48.25, respectively in the final wave (wave 16).

### Regression models

Table 4 demonstrates the relationships between different levels of cognitive impairment and the HRQoL outcome measures (PCS, MCS, SF-6D) for this study. The regression coefficients of the cognitive impairment concerning the PCS, MCS, and SF-6D are displayed in models 1 to 3. Model 1 shows that PCS scores of those who were severely cognitively impaired scored over 3 unit points (β = − 3.560) lower than normal, while PCS scores of those with moderate cognitive impairment scored almost 2 unit points (β = − 1.765) lower than normal. Model 2 shows that MCS scores of those who were severely cognitively impaired scored over 1 unit point (β = − 1.759) lower than normal, but could not significantly demonstrate this. Conversely, Model 2 demonstrates a significant decrease in MCS scores compared to normal for those who are moderately cognitively impaired, showing a decrease of almost 2 unit points (β = − 1.612) lower than normal. Finally, model 3 shows that SF-6D utility scores of those who were severely cognitively impaired scored over 0.03 unit points (β = − 0.034) lower than normal, while SF-6D utility scores of those with moderate cognitive impairment scored over 0.02 unit points (β = − 0.024) lower than normal.

**Table 4:** The association between cognitive impairment and the SF–36 component summary scores and SF–6D utility score, a longitudinal random-effects analysis

Variables	Model 1: PCS	Model 2: MCS	Model 3: SF-6D
	β [SE]	β [SE]	β [SE]
Cognitive impairment			
No (ref)			
Moderate	− 1.765***	− 1.612***	− 0.024***
	[0.317]	[0.326]	[0.004]
Severe	− 3.560**	− 1.759	− 0.034**
	[1.103]	[1.289]	[0.012]
Age			
50–64 years (ref)			
≥ 65 years	− 1.707***	3.604***	0.016***
	[0.225]	[0.242]	[0.003]
Gender			
Male (ref)			
Female	− 0.629**	0.004	− 0.007**
	[0.196]	[0.223]	[0.002]
Relationship status			
Single (ref)			
Couple	0.680***	1.205***	0.016***
	[0.202]	[0.231]	[0.002]
Highest education level completed			
Year 12 and below (ref)			
Certificate courses	0.255	− 0.101	− 0.002
	[0.228]	[0.258]	[0.003]
University degrees	0.922***	− 0.373	0.001
	[0.253]	[0.290]	[0.003]
Household annual disposable income			
Quintile 1 (Poorest)	− 2.235***	− 1.230***	− 0.025***
	[0.301]	[0.331]	[0.004]
Quintile 2 (Poor)	− 1.683***	− 0.915**	− 0.019***
	[0.272]	[0.300]	[0.003]
Quintile 3 (Middle)	− 0.899***	− 0.312	− 0.008**
	[0.246]	[0.275]	[0.003]
Quintile 4 (Richer)	− 0.428	− 0.070	− 0.003
	[0.227]	[0.258]	[0.003]
Quintile 5 (Richest) (ref)			
Labour force status			
Employed (ref)			
Unemployed or NLF	− 2.742***	− 1.443***	− 0.032***
	[0.223]	[0.250]	[0.003]
Indigenous status			
Not of Indigenous origin (ref)			
Indigenous origin	0.086	0.480	0.006
	[0.701]	[0.840]	[0.009]
Region of residence			
Major city (ref)			
Regional or remote area	− 0.679***	0.435*	− 0.001
	[0.199]	[0.218]	[0.002]
Smoking status			
Non–smoker (ref)			
Current smoker	− 0.505	− 2.171***	− 0.016***
	[0.283]	[0.335]	[0.003]
Alcohol consumption			
Non–drinker (ref)			
Current drinker	1.389***	1.142***	0.017***
	[0.259]	[0.280]	[0.003]
Physical Activity			
Less than the recommended level (ref)			
Recommended level	2.486***	2.128***	0.035***
	[0.177]	[0.181]	[0.002]
BMI			
Underweight	− 0.710	− 2.772**	− 0.021*
	[0.762]	[0.929]	[0.009]
Healthy weight (ref)			
Overweight	− 0.900***	0.013	− 0.007**
	[0.202]	[0.219]	[0.002]
Obese	− 3.342***	− 0.575*	− 0.026***
	[0.241]	[0.260]	[0.003]
Disability			
No (ref)			
Yes	− 9.051***	− 3.146***	− 0.085***
	[0.211]	[0.208]	[0.002]

Notes: 1. Robust standard errors are reported in square brackets

2. Abbreviations: *ref* = reference category, *PCS* = Physical Component Summary, *MCS* = Mental Component Summary, *SF-6D* = Short-Form Six-Dimension health utility index

3. Significance level: ***p < 0.001, **p < 0.01, *p < 0.05

## Discussion

This study examined the association between cognitive impairment with HRQoL in a sample of older Australians, using data from the HILDA survey. The findings indicated that majority of older Australians do not have cognitive impairment (89%). However, 10% and 1% had moderate and severe cognitive impairment, respectively. Similar levels of cognitive impairment amongst older Australians (aged ≥ 65 years) have been documented elsewhere [8, 9].

We measured HRQoL by the generic non-preference based measure (SF-36) and generic preference based measure (SF-6D). Generic tools have been criticised for not being sensitive for cognition. However, our results indicate that possibly SF-36 is a suitable tool for measuring change in quality of life related to cognitive status. The study showed strong evidence of an association between moderate and severe cognitive impairment with lower HRQoL concerning physical function (PCS) and the SF-6D. With regards to mental function (MCS), there was a strong association between moderate cognitive impairment and lower HRQoL after adjusting for confounding factors. A similar association between cognitive impairment and low HRQoL has been observed in older community-based participants from Australia [40], the United States [40], Japan [41], China [20] and Sweden [21]. Cognitive impairment has been associated with lower ratings of physical and mental components of HRQoL [20, 21]. Amongst community-dwelling older people, a 10-unit higher PCS and MCS score was associated with a 12% and 6% decreased risk of cognitive impairment, respectively [40]. Alternatively, no association was found between cognitive impairment and HRQoL in a community-dwelling sample of older adults [28]. However, this was attributed to the use of EuroQOL (EQ-5D) to measure HRQoL which does not contain a domain related to cognition, and thus may not be well associated with the cognitive impairment screening measure (i.e., Montreal Cognitive Assessment) used in the study [42, 43]. This issue has been previously debated [44]. Furthermore, as demonstrated in this study, severe cognitive impairment was associated with lower HRQoL scores compared to mild cognitive impairment in other studies [21, 29]. The concordance in community-based findings across various samples and this study’s findings add weight to the association between cognitive impairment and HRQoL.

Additionally, this study revealed differing strengths in associations between the mental and physical components of HRQoL with cognitive impairment. MCS and PCS scores assess unique aspects of self-reported HRQoL, which may account for differing associations with cognitive impairment. The current study illustrated that cognitive impairment was negatively associated with PCS and MCS, albeit the negative association between severe cognitive impairment and lower MCS scores was not statistically significant. In contrast, previous research on healthy older adults have shown differing results on the mental and physical components of HRQoL. A cross-sectional study on healthy older adults (aged 55 and older) showed a negative association between the physical component of HRQoL with age but a positive association between the mental component of HRQoL with age [45]. This is in line with theoretical views that older adults may maintain mental well-being despite objective health losses given that they possess self-regulatory mechanisms [46].

A plausible reason for the association between cognitive impairment and low HRQoL is the perception of poor health. The deterioration of HRQoL amongst the cognitively impaired elderly has been primarily attributed to loss of autonomy, which affects the ability to complete basic daily tasks independently [47, 48]. Cognitively impaired individuals may experience frustrations from the inability to complete basic daily tasks independently [48]. Therefore, individuals may experience heightened depression, anxiety, and dysfunctional social interactions [49], which may further deteriorate HRQoL. Prior studies have highlighted that older adult with cognitive impairment has a higher likelihood of reporting problems in HRQoL dimensions such as pain/discomfort, and anxiety/depression [20, 50]. Thus, it has been argued that an awareness of cognitive impairment may lead to distress as a reaction to declining cognition in elderly adults [50]. Independent of the severity of cognitive impairment, older adults aware of a mild cognitive impairment diagnosis and its prognosis had lower HRQoL, and more difficulties with daily functioning than those who were unaware [51]. Conversely, if individuals with cognitive impairment do not perceive their functioning as impaired, they may report a non-deteriorated HRQoL. Given that individuals assess their HRQoL by comparing their expectations and experience, perceived functional limitations may lower HRQoL [52]. Therefore, promoting practical coping strategies could improve HRQoL among cognitively impaired older adults. However, as cognitive impairment is associated with functional decline and multiple morbidities [53], it may curtail compensatory mechanisms amongst cognitively impaired elderly to maintain good mental well-being in contrast to their healthier, community-dwelling counterparts.

This study adds to the existing evidence that cognitive impairment is associated with low HRQoL in older adults. The findings highlight the importance of sustaining the cognitive ability to improve HRQoL in healthy ageing, particularly for the physical component of HRQoL. This presents a public health opportunity for policymakers to design and implement strategies that will maintain cognitive functioning or relieve symptoms associated with cognitive decline. It is hoped that such efforts may meaningfully improve HRQoL and potentially prevent individuals from declining further into severe cognitive impairment seen in dementia.

As cognitive decline is associated with functional impairments, this may pose difficulties in fulfilling basic needs, thus leading to poor physical and mental health. Specifically, assisting cognitively impaired older adults with feelings of loneliness, pain and improving the ability to undertake basic activities of daily living may have important implications for mental and physical HRQoL [54–56]. Therefore, promoting purposeful care to address a broad range of modifiable risk factors and encouraging protective factors may be key. These include social engagement, cognitive and physical activity and may serve as an effective strategy to maintain HRQoL with advancing age [57–59]. Besides, there is evidence that a patient empowerment model that considers the patient as the prime member of the health team and care managers who provide services to patients suggested by physicians in the primary health care system is beneficial for improving health outcomes for patients with heart failure and diabetes [60]. Hence, introducing a patient empowerment model might be helpful to reduce cognitive impairment among older Australians. Moreover, the literature has highlighted the importance of preventative measures in sustaining cognitive function. Recent evidence illustrated that 21.7% of MCI cases that deteriorated in dementia may have been preventable by targeting diet (8.7%), diabetes (1.5%) and neuropsychiatric symptoms (11.5%) [61]. Furthermore, specific lifestyle factors such as engaging in social and artistic activities during midlife and late were protective against cognitive impairment at ages 85–89 [62]. This evidence reinforces the need for further research to broadly understand health status across multiple domains, and formulate preventative measures against cognitive deterioration. Thus, by understanding the overall impact of cognitive impairment on HRQoL, cognitive measures may be utilised as a pragmatic tool to identify those who may benefit from such preventative strategies.

A key contribution of this study is the suggested cut-off scores to define cognitive impairment. To the authors’ knowledge, there is limited literature on appropriate cut-off scores that define cognitive impairment using the BDS and SDMT in older adults. BDS cut-off scores ≤ 4 have been used as a threshold to define cognitive impairment in older adults [36, 63, 64]. Previous research has suggested SDMT cut-off scores between 24 and 40 to define cognitive impairment. However, such research was based on highly specific groups, particularly, those with multiple sclerosis [34, 65–67]. The generated cut-off scores used in the current study generally align with previous literature discussed previously. Nevertheless, the current study suggested a SDMT cut-off score of ≤ 30, which lies in the range suggested by previous studies as discussed. However, the BDS cut-off score of ≤ 3 was more conservative than previous literature, and thus may be less sensitive in capturing milder cognitive impairment. Nevertheless, it is hoped that the suggested cut-off scores for cognitive impairment in the current study may contribute to the limited body of literature on optimal cut-off scores for defining cognitive impairment in the elderly. Much of the previous literature has focused on highly specific samples, yielding inconsistent findings on the association between cognitive impairment and HRQoL. This may be attributed to the heterogeneity in study samples, settings, and methodology. Furthermore, to the authors’ knowledge, majority of studies assessing the association between cognitive impairment and HRQoL have been cross-sectional in nature. Thus, no causative association could be firmly established. By undertaking this longitudinal study in a nationally representative cohort of older Australians, findings on the impact of cognitive impairment on HRQoL may be more generalisable to the normal ageing process and may be applied to future research and policy endeavours.

### Strength and weaknesses

A major strength of this study was its large, population-based longitudinal nature, covering a wide spectrum of older age (50 years and older). This study utilised 10,737 person-year observations from 6892 unique individuals, using two waves of the HILDA survey to examine the association between cognitive impairment and HRQoL amongst older Australians. Further, a variety of factors were controlled for, and findings remained robust with adjustment. However, given the design of the study, unmeasured and unknown confounders could exist and impact the results.

The cognitive impairment measures (SDMT and BDS) are also validated and have good utility in reflecting core constructs of cognitive ageing and impairment. The use of validated tools facilitates the comparison of this study’s findings with previous research. A notable limitation of this study was the data collection methods for HRQoL. As HRQoL was self-reported, there is a risk of social desirability bias, leading to an inflation of HRQoL scores. Secondly, there is no consensus on clear cut-off scores for the SDMT and BDS scales to define cognitive impairment. Therefore, these measures are not diagnostic of cognitive impairment and may not accurately capture a full spectrum of cognitive impairment that is clinically significant. Thirdly, chronic conditions and pharmacological treatment are two crucial confounding variables in the relationship between cognitive impairment and HRQoL. Due to the unavailability of chronic conditions and pharmacologic data, the authors can’t include these confounding factors in the multivariate regression. Therefore, the estimated co-efficient could be underestimated or overestimated.

## Conclusion

This study examines the association between cognitive impairment and HRQoL in older Australian adults. We found that those deemed mildly and severely cognitively impaired had lower PCS, and SF-6D scores than those with no cognitive impairment. Our findings might be helpful for performing future economic evaluation aiming to reduce cognitive impairment as the results revealed the disutility associated with moderate and severe cognitive impairment. Given that this study found a strong association between mild cognitive impairment and the physical component, but not the mental component of HRQoL, further research may be needed to understand how these components may be differentially affected by cognitive impairment across a spectrum of old age. Such understanding may be leveraged to promote targeted interventions that prevent or alleviate symptoms of poor HRQoL in those with cognitive impairment. Along similar lines, future studies could systematically evaluate specific elements of cognitive functioning to better understand their relative impact on HRQoL. This may assist in identifying preventative strategies to improve specific cognitive functions to yield a greater degree of improvement in HRQoL as adults enter older age.

## Data Availability

The data were obtained from the Melbourne Institute of Applied Economic and Social Research (https://melbourneinstitute.unimelb.edu.au/). Though the information is not available openly, one can access the data after accepting their guidelines and restrictions. The contact address is Melbourne Institute of Applied Economic and Social Research, the University of Melbourne, VIC 3010, Australia.

## Abbreviations

EQ-5D: European Quality of Life Five Dimension
HILDA: Household, Income and Labour Dynamics in Australia
HRQoL: Health-related quality of life
PCS: Physical component summary
MCS: Mental component summary
SF-6D: Short-Form Six-Dimension
SF-36: 36-Item Short-Form Health Survey

## References

[1] Pais R, Ruano L, Moreira C, Carvalho OP, Barros H (2020). Prevalence and incidence of cognitive impairment in an elder Portuguese population (65–85 years old). BMC Geriatrics.

[2] AIHW (2021). Older Australians.

[3] Nilsson L-G, Sternäng O, Rönnlund M, Nyberg L (2009). Challenging the notion of an early-onset of cognitive decline. Neurobiology of Aging.

[4] Finch CE (2009). The neurobiology of middle-age has arrived. Neurobiology of Aging.

[5] Salthouse TA (2009). When does age-related cognitive decline begin?. Neurobiology of Aging.

[6] Hedden T, Gabrieli JDE (2004). Insights into the ageing mind: A view from cognitive neuroscience. Nature Reviews Neuroscience.

[7] Singh-Manoux A, Kivimaki M, Glymour MM, Elbaz A, Berr C, Ebmeier KP, Dugravot A (2012). Timing of onset of cognitive decline: results from Whitehall II prospective cohort study. BMJ.

[8] Low L-F, Brodaty H, Low L-F, Brodaty H, Edwards R, Kochan N, Sachdev P (2004). The Prevalence of ‘cognitive impairment no dementia’ in community-dwelling elderly: A pilot study. Australian & New Zealand Journal of Psychiatry.

[9] Anderson TM, Sachdev PS, Brodaty H, Trollor JN, Andrews G (2007). Effects of sociodemographic and health variables on mini-mental state exam scores in older Australians. The American Journal of Geriatric Psychiatry.

[10] Pais R, Ruano L, Carvalho P, Barros H (2020). Global cognitive impairment prevalence and incidence in community dwelling older adults—A systematic review. Geriatrics.

[11] WHO (2019). Risk reduction of cognitive decline and dementia: WHO guidelines.

[12] WHO (2017). Global action plan on the public health response to dementia 2017–2025.

[13] VandenBos GR (2007). APA Dictionary of Psychology.

[14] Harada CN, Natelson Love MC, Triebel KL (2013). Normal cognitive aging. Clinics in Geriatric Medicine.

[15] Roberts R, Knopman DS (2013). Classification and Epidemiology of MCI. Clinics in Geriatric Medicine.

[16] Geda YE (2012). Mild cognitive impairment in older adults. Current Psychiatry Reports.

[17] Bowling A (2001). Measuring disease.

[18] Muller AE, Skurtveit S, Clausen T (2016). Validating the generic quality of life tool “QOL10” in a substance use disorder treatment cohort exposes a unique social construct. BMC Medical Research Methodology.

[19] Lorgelly PK, Doble B, Rowen D, Brazier J (2017). Condition-specific or generic preference-based measures in oncology? A comparison of the EORTC-8D and the EQ-5D-3L. Quality of Life Research.

[20] Pan C-W, Wang X, Ma Q, Sun H-P, Xu Y, Wang P (2015). Cognitive dysfunction and health-related quality of life among older Chinese. Scientific Reports.

[21] Johansson MM, Marcusson J, Wressle E (2012). Cognition, daily living, and health-related quality of life in 85-year-olds in Sweden. Aging, Neuropsychology, and Cognition.

[22] Akdag B, Telci EA, Cavlak U (2013). Factors affecting cognitive function in older adults: A Turkish sample. International Journal of Gerontology.

[23] Livingston G, Cooper C, Woods J, Milne A, Katona C (2007). Successful ageing in adversity: The LASER-AD longitudinal study. Journal of Neurology, Neurosurgery & Psychiatry.

[24] van der Zon A, Wetzels RB, Bor H, Zuidema SU, Koopmans RTCM, Gerritsen DL (2018). Two-year course of quality of life in nursing home residents with dementia. The American Journal of Geriatric Psychiatry.

[25] Mitchell AJ, Kemp S, Benito-León J, Reuber M (2010). The influence of cognitive impairment on health-related quality of life in neurological disease. Acta Neuropsychiatrica.

[26] Elliott AF, McGwin G, Owsley C (2009). Health-related quality of life and visual and cognitive impairment among nursing-home residents. British Journal of Ophthalmology.

[27] Banerjee S, Samsi K, Petrie CD, Alvir J, Treglia M, Schwam EM, del Valle M (2009). What do we know about quality of life in dementia? A review of the emerging evidence on the predictive and explanatory value of disease specific measures of health related quality of life in people with dementia. International Journal of Geriatric Psychiatry.

[28] Davis JC, Bryan S, Li LC, Best JR, Hsu CL, Gomez C, Liu-Ambrose T (2015). Mobility and cognition are associated with wellbeing and health related quality of life among older adults: a cross-sectional analysis of the Vancouver Falls Prevention Cohort. BMC Geriatrics.

[29] Missotten P, Squelard G, Ylieff M, Di Notte D, Paquay L, De Lepeleire J, Fontaine O (2008). Quality of life in older Belgian people: Comparison between people with dementia, mild cognitive impairment, and controls. International Journal of Geriatric Psychiatry.

[30] Watson N (2021). Finding your way around the HILDA survey data. Australian Economic Review.

[31] Brazier JE, Harper R, Jones NM, O’Cathain A, Thomas KJ, Usherwood T, Westlake L (1992). Validating the SF-36 health survey questionnaire: New outcome measure for primary care. BMJ.

[32] Australian Bureau of Statistics (1997). 1995 National health survey—SF-36 population norms, Australia.

[33] Ferreira LN, Ferreira PL, Pereira LN, Rowen D, Brazier JE (2013). Exploring the consistency of the SF-6D. Value in Health.

[34] Van Schependom J, Dhooghe MB, Cleynhens K, Dhooge M, Haelewyck MC, De Keyser J, Nagels G (2014). The symbol digit modalities test as sentinel test for cognitive impairment in multiple sclerosis. European Journal of Neurology.

[35] Parmenter BA, Weinstock-Guttman B, Garg N, Munschauer F, Benedict RH (2007). Screening for cognitive impairment in multiple sclerosis using the symbol digit modalities test. Multiple Sclerosis Journal.

[36] Leung JLM, Lee GTH, Lam YH, Chan RCC, Wu JYM (2011). The use of the digit span test in screening for cognitive impairment in acute medical inpatients. International Psychogeriatrics.

[37] Lamar M, Price CC, Libon DJ, Penney DL, Kaplan E, Grossman M, Heilman KM (2007). Alterations in working memory as a function of leukoaraiosis in dementia. Neuropsychologia.

[38] Smith A (1973). Symbol digit modalities test.

[39] Aschwanden D, Sutin AR, Luchetti M, Stephan Y, Terracciano A (2020). Personality and dementia risk in England and Australia. GeroPsych.

[40] Phyo AZZ, Gonzalez-Chica DA, Stocks NP, Storey E, Woods RL, Murray AM, Ryan J (2021). The utility of assessing health-related quality of life to predict cognitive decline and dementia. Journal of Alzheimer’s Disease.

[41] Kitamura K, Nakamura K, Ueno K, Nishiwaki T (2016). Cognitive function is maintained in noninstitutionalized elderly Japanese requiring care with high levels of health-related quality of life. Environmental Health and Preventive Medicine.

[42] Rabin R, de Charro F (2001). EQ-SD: A measure of health status from the EuroQol Group. Annals of Medicine.

[43] Nasreddine ZS, Phillips NA, Bacdirian V, Charbonneau S, Whitehead V, Collin I, Chertkow H (2005). The montreal cognitive assessment, MoCA: A brief screening tool for mild cognitive impairment. Journal of the American Geriatrics Society.

[44] Couzner L, Crotty M, Norman R, Ratcliffe J (2013). A Comparison of the EQ-5D-3L and ICECAP-O in an older post-acute patient population relative to the general population. Applied Health Economics and Health Policy.

[45] Tseng H-Y, Löckenhoff C, Lee C-Y, Yu S-H, Wu I-C, Chang H-Y, Hsiung CA (2020). The paradox of aging and health-related quality of life in Asian Chinese: Results from the healthy aging longitudinal study in Taiwan. BMC Geriatrics.

[46] Albrecht GL, Devlieger PJ (1999). The disability paradox: High quality of life against all odds. Social Science & Medicine.

[47] Andersen CK, Wittrup-Jensen KU, Lolk A, Andersen K, Kragh-Sørensen P (2004). Ability to perform activities of daily living is the main factor affecting quality of life in patients with dementia. Health and Quality of Life Outcomes.

[48] Rickenbach EH, Condeelis KL, Haley WE (2015). Daily stressors and emotional reactivity in individuals with mild cognitive impairment and cognitively healthy controls. Psychology and Aging.

[49] Hill NL, McDermott C, Mogle J, Munoz E, DePasquale N, Wion R, Whitaker E (2017). Subjective cognitive impairment and quality of life: A systematic review. International Psychogeriatrics.

[50] Vinkers DJ, Gussekloo J, Stek ML, Westendorp RGJ, van der Mast RC (2004). Temporal relation between depression and cognitive impairment in old age: Prospective population based study. BMJ.

[51] Stites SD, Karlawish J, Harkins K, Rubright JD, Wolk D (2017). Awareness of mild cognitive impairment and mild Alzheimer’s disease dementia diagnoses associated with lower self-ratings of quality of life in older adults. The Journals of Gerontology: Series B.

[52] Carr AJ (2001). Measuring quality of life: Is quality of life determined by expectations or experience?. BMJ.

[53] Calderón-Larrañaga A, Vetrano DL, Ferrucci L, Mercer SW, Marengoni A, Onder G, Fratiglioni L (2019). Multimorbidity and functional impairment-bidirectional interplay, synergistic effects and common pathways. Journal of Internal Medicine.

[54] Christiansen L, Sanmartin Berglund J, Lindberg C, Anderberg P, Skär L (2019). Health-related quality of life and related factors among a sample of older people with cognitive impairment. Nursing Open.

[55] Chan C, Slaughter S, Jones C, Wagg A (2015). Greater independence in activities of daily living is associated with higher health-related quality of life scores in nursing home residents with dementia. Healthcare.

[56] Logsdon RG, Gibbons LE, McCurry SM, Teri L (2002). Assessing quality of life in older adults with cognitive impairment. Psychosomatic Medicine.

[57] Williams KN, Kemper S (2010). Interventions to reduce cognitive decline in aging. Journal of Psychosocial Nursing and Mental Health Services.

[58] Alsubaie SF, Alkathiry AA, Abdelbasset WK, Nambi G (2020). The physical activity type most related to cognitive function and quality of life. BioMed Research International.

[59] Lee SH, Kim YB (2016). Which type of social activities may reduce cognitive decline in the elderly?A longitudinal population-based study. BMC Geriatrics.

[60] Ciccone M, Bux F, Cortese A, Pomo M, Scicchitano P (2010). Feasibility and effectiveness of a disease and care management model in the primary health care system for patients with heart failure and diabetes (Project Leonardo). Vascular Health and Risk Management.

[61] Livingston G, Huntley J, Sommerlad A, Ames D, Ballard C, Banerjee S, Mukadam N (2020). Dementia prevention, intervention, and care: 2020 report of the lancet commission. The Lancet.

[62] Roberts RO, Cha RH, Mielke MM, Geda YE, Boeve BF, Machulda MM, Petersen RC (2015). Risk and protective factors for cognitive impairment in persons aged 85 years and older. Neurology.

[63] Legdeur N, Binnekade TT, Otten RH, Badissi M, Scheltens P, Visser PJ, Maier AB (2017). Cognitive functioning of individuals aged 90 years and older without dementia: A systematic review. Ageing Research Reviews.

[64] Muangpaisan W, Intalapaporn S, Assantachai P (2010). Digit span and verbal fluency tests in patients with mild cognitive impairment and normal subjects in Thai-community. Journal of the Medical Association of Thailand = Chotmaihet thangphaet.

[65] Sepulcre J, Vanotti S, Hernández R, Sandoval G, Cáceres F, Garcea O, Villoslada P (2006). Cognitive impairment in patients with multiple sclerosis using the brief repeatable battery-neuropsychology test. Multiple Sclerosis Journal.

[66] Meca-Lallana V, Gascón-Giménez F, Ginestal-López RC, Higueras Y, Téllez-Lara N, Carreres-Polo J, Pérez-Miralles F (2021). Cognitive impairment in multiple sclerosis: Diagnosis and monitoring. Neurological Sciences.

[67] Benedict RH, DeLuca J, Phillips G, LaRocca N, Hudson LD, Rudick R (2017). Validity of the symbol digit modalities test as a cognition performance outcome measure for multiple sclerosis. Multiple Sclerosis Journal.

